# Calcium-Binding Protein and Polymorphism in *Musa* spp. Somaclones Resistant to *Fusarium oxysporum*

**DOI:** 10.3390/cimb46110719

**Published:** 2024-10-29

**Authors:** Juliana Rodrigues Sampaio, Wanderley Diaciso dos Santos Oliveira, Fernanda dos Santos Nascimento, Luiz Carlos de Souza Junior, Tamyres Amorim Rebouças, Ricardo Franco Cunha Moreira, Andresa Priscila de Souza Ramos, Janay Almeida dos Santos-Serejo, Edson Perito Amorim, Claudia Fortes Ferreira

**Affiliations:** 1 Department of Agricultural, Environmental and Biological Sciencies, Federal University of Recôncavo da Bahia, Rua Rui Barbosa, 710-Centro, Cruz das Almas 44380-000, BA, Brazil; sampaiorodriguesjuliana@gmail.com (J.R.S.); juniorluiz6666@gmail.com (L.C.d.S.J.); ricardofcm@ufrb.edu.br (R.F.C.M.); 2 Department of Biological Sciences, Feira de Santana State University, Feira de Santana 44036-900, BA, Brazil; diacisowanderley@hotmail.com; 3 Embrapa Mandioca e Fruticultura, Rua Embrapa, s/no, Chapadinha, Cruz das Almas 44380-000, BA, Brazil; feel.20@hotmail.com (F.d.S.N.); tamyres.amorim@yahoo.com.br (T.A.R.); andresa.ramos@embrapa.br (A.P.d.S.R.); janay.serejo@embrapa.br (J.A.d.S.-S.); edson.amorim@embrapa.br (E.P.A.)

**Keywords:** Foc ST4, retrotransposons, IRAP, REMAP, BLAST

## Abstract

The fresh fruits of ‘Grande Naine’ (Cavendish AAA—*Musa* spp.) dominate the world market, especially in countries with a population in a situation of social vulnerability. However, Fusarium wilt, caused by the fungus *Fusarium oxysporum* f.sp. *cubense* race 4 Subtropical (Foc ST4), emerges as a serious threat to banana production, requiring the development of resistant cultivars based on biotechnological strategies, such as the induction of mutation in tissue culture. This study aimed to identify and characterize genetic variation in somaclones resistant to *Fusarium oxysporum* f.sp. *cubense* subtropical race 4 (Foc ST4), derived from ‘Grand Naine’ bananas, by molecular markers based on retrotransposons IRAP (Inter-retrotransposon Amplified Polymorphism) and REMAP (Retrotransposon-Microsatellite Amplified Polymorphism). Nine combinations of IRAP and six combinations of REMAP primers were used. The low number of polymorphic bands did not allow for genetic diversity studies; however, ten polymorphic bands between the somaclones and control were sequenced. Of these, three presented good base calling and were aligned, namely, 1AF, 2AF, and 3AF bands. Only the 1AF band presented function related to stress response with homology to a calcium-binding protein. These proteins act early in plant infection as secondary messengers activated by pathogen-associated molecular patterns (PAMPs), initiating the cascade of plant defense signals. The fact that this band is present in all somaclones reinforces previous assessments of their resistance to Foc ST4. The use of markers IRAP and REMAP produced polymorphic bands that can, through future primer design and field validations, accelerate the identification of resistant banana genotypes for use in banana genetic breeding programs.

## 1. Introduction

Originating in Southeast Asia, bananas, *Musa* spp., are one of the most planted fruits of tropical and subtropical regions [1,2,3], especially those from the Cavendish subgroup (AAA), an important commodity in the global market and staple food for socially vulnerable populations [3,4]. In 2022, global banana production reached approximately 135 million tons in an area of 5.9 million hectares, with Asia accounting for 51.8% of total production, followed by the Americas (23.8%) and Africa (22.8%) [1].

This wide geographical distribution and adaptation of bananas in different environments resulted in varied interactions between their genotypes and local pathogens. However, the *Musa* genus has a very narrow genetic background regarding resistance-related genes [1], and commonly cultivated bananas are susceptible, with different levels of tolerance or resistance to bacterial, viral, and fungal diseases [5,6,7]. Among fungal diseases, Fusarium wilt caused by the fungus *Fusarium oxysporum* f. sp. *cubense*, (E.F. Smith) W.C. Snyder and H.N. Hansen (Foc), races 1, 2, and 4 are very destructive, whereas the latter causes the most damage [8,9]. The fact that Foc spores can stay in the soil for many years in the form of clamydospores aggravates the situation in banana plantations worldwide. There is no chemical control for Foc, and resistant varieties are still the main means of control. This disease impacts banana productivity, leading, in some cases, to the complete loss of production [10,11], in addition to economically and environmentally burdening disease mitigation actions, making it impossible for small producers to continue in the activity and, thus, aggravating poverty [9,12,13,14].

The Cavendish subgroup is resistant to Fusarium wilt caused by race 1. However, it is susceptible, as are all commercial triploids, to tropical race 4 (Foc TR4) and subtropical race 4 (Foc ST4), which differ in the severity of symptoms and existence of a conditioning factor, low temperatures, and water stress for the development of the disease caused by Foc ST4 [11,15].

Bananas are parthenocarpic fruits, or have a low seed count with viable seeds, a condition endemic to the species, which eventually leads to a narrow genetic background. Genetic somaclonal variation is an alternative source of variability [16,17] and may occur due to pre-existing conditions or related to the specificities of in vitro tissue culture [18]. These variations consist of a set of phenotypically expressed changes caused by induced stress [19,20,21] or resulting from the many sub-cultivations during the in vitro multiplication process [22,23] and can result in changes in the DNA sequence, genes, or chromosomes being stable and heritable [23] and can genetically differentiate derived somaclones.

Retrotransposon-based DNA molecular markers are promising tools for detecting somaclonal variation due to the close relationship between stress, biotic or abiotic [24], and the activation of transposable elements in plant DNA. Its applicability is related to its displacement characteristic, “copy and paste” along the DNA strand, creating copies of itself by a transcription mechanism, which can be inserted in different loci [25]. Thus, their new insertions are traces detectable with target amplification mechanisms using polymerase chain reactions, enabling studies of genetic variability and diversity in the genus *Musa* [26,27,28].

Additionally, their movement can result in insertion in regions close to genes, and their regulatory and signaling elements can interfere with the expression pattern of these genes, as well as inactivate them by silencing with methylation action [29]. Inter-retrotransposon amplified polymorphism (IRAP) and retrotransposon-microsatellite amplified polymorphism (REMAP) are two dominant methods of DNA polymorphism detection, based on retroelements, that are widely used [24,30].

The IRAP marker method was developed from the proximity between two LTRs (Long Terminal Repeats) [31] and amplifies the region between the final LTR portion of one retrotransposon and the other. The REMAP marker identifies intrageneric and intraspecific polymorphisms and amplifies the region between the LTR portion and the next microsatellite. Research using IRAP and REMAP markers has been conducted, aiming, for example, to evaluate the variability and genetic structure of populations of *Musa* spp., *Medicago polymorpha*, and *Avena sativa* [28,32,33]; the transfer and reproducibility of markers between *Piper nigrum* species [34] and the analysis of intravarietal diversity and between cultivars and local varieties of *Vitis vinifera* [35,36].

In general, the molecular approach with IRAP and REMAP provides evidence of the activation of these markers, with high polymorphism [37,38]. In some cases, these markers are correlated with phenotypic characteristics [37,39], as reported by Rashid et al. [40], as they show a strong correlation between the IRAP marker and the phenotypic characteristic of resistance of *Carica papaya* accessions to the mosaic virus.

In tissue culture, the polymorphism and, in some cases, its relationship with the response of plants to abiotic and biotic stress was investigated. Shingote et al. [41] used 32 IRAP markers and 100 ISSR markers in the clonal fidelity test in 47 accessions of *Saccharum* spp., with an IRAP marker identifying a clonal variant whose unique bands were confirmed with sequencing. Mirany et al. [42] investigated the somaclonal variation in date palms, detecting a high percentage of similarity between the regenerators and the mother plant. In the study conducted by Nasri et al. [43], the IRAP and ISSR markers made it possible to identify chrysanthemum mutants as a function of the cultivar and the characteristic of the chemical inducer. Arvas et al. [24] demonstrated, with IRAP and REMAP markers, the differentiated activation of retroransposons in genomic DNA extracted from different mutant and non-mutant parts of *Oryza* spp. in response to salt stress in tissue culture. Muhammad and Othman [44] evaluated mutant somaclones resistant and susceptible to *Fusarium oxysporum* f. sp. cubense race 4, identifying polymorphism with IRAP and random amplified polymorphic DNA (RAPD) markers without, however, identifying specific bands that were exclusive to the clones of the cultivars Rastali (susceptible) or Mutiara (resistant).

In studies on somaclonal variation in bananas, although extensive, few report the sequencing of polymorphic bands between resistant and susceptible genotypes in order to evaluate the ability to aggregate information to the polymorphic individual. In our study, ‘Grande Naine’ (GN) somaclones, induced by phytoregulators thidiazuron (TDZ) and paclobutrazol (PBZ), previously infected by a virulent Foc ST4 isolate—CNPMF218A- and considered resistant, were selected and compared to the GN mother plant with retrotransposon markers. Thus, this study aimed to identify and characterize somaclonal variation in plants derived from the cultivar Grande Naine (Cavendish—AAA), previously considered resistant to Foc STR4 compared to its control (mother plant moderately resistant), using two retrotransposon-based molecular marker systems, IRAP and REMAP.

## 2. Materials and Methods

### 2.1. Plant Material

Twelve plants resistant to Foc ST4, derived from the cultivar Grande Naine (Cavendish—AAA, *Musa* spp.), belonging to the Banana Germplasm Bank (BAG-Banana) of Embrapa Mandioca e Fruticultura, located in Cruz das Almas, Bahia, Brazil, were evaluated. To identify somaclonal variation, the plant derived from the commercial cultivar Grande Naine, moderately resistant to Foc ST4, namely “Control”, was also evaluated, allowing the comparison between the somaclones and between them and the cultivar of origin (Control).

The resistant somaclones evaluated were obtained in a previous study by inducing somaclonal variation, according to the methodology used by Rebouças et al. [21] with a subculture of shoot apices in Murashige and Skoog medium enriched with the phytoregulators thidiazuron (TDZ) and paclobutrazol (PBZ). The selection of resistant somaclones was performed with controlled inoculation in a greenhouse with the CNPMF218A isolate, corresponding to Foc ST4. The 14 surviving somaclones without symptoms (Embrapa 4 and Embrapa 17) were micropropagated, acclimated, and planted in an experimental field of Embrapa Mandioca e Fruticultura for agronomic evaluations. Of these 14 micropropagated somaclones that survived, four of them were pre-selected for evaluation in this study.

At the time of this study, the somaclones micropropagated were established in the field in their first production cycle, when three clones of each of the four treatments evaluated (T1—Embrapa 14, T2—Embrapa 15, T3—Embrapa 16, and T4—Embrapa 17) were randomly selected, making up, together with the control plant, the sample set (Table 1).

### 2.2. Extraction and Quantification of Genomic DNA

Fresh cuts of young leaves (300 mg) were collected from each plant, and DNA was extracted and purified following the modified CTAB protocol by Ferreira et al. [45]. The leaf tissues were macerated with the aid of an adapted bench drill in the presence of 3 mL of CTAB extraction buffer (2.4%) containing EDTA (20 mM), NaCl (1.7 M), Tris-HCl pH 8.0 (0.1 M), PVP (2%) and β-mercaptoethanol (0.4%). DNA samples were standardized at a concentration of 10 ng·µL^−1^.

### 2.3. Polymerase Chain Reaction and Molecular Characterization

For the detection of somaclonal variation, two multilocus techniques based on polymerase chain reaction (PCR) were employed. For analysis using the PCR-IRAP (Inter-Retrotransposon Amplified Polymorphism) technique, eight LTR (long terminal repeat) primers [26,46] from different families were paired (Table 2).

For this work, only the markers that generated electrophoretic profiles with good resolution, with easily visualized bands, were considered. The same procedure was adopted for the other reactions and marker systems.

The markers used in the PCR-REMAP (Retrotransposon-Microsatellite Amplified Polymorphism) technique were obtained from the paired combination between an LTR-type retrotransposon primer and one of the six microsatellite primers (SSR—single simple repeat) [31], as shown in Table 3.

PCR-IRAP and PCR-REMAP amplification reactions were performed with a final volume of 15 µL per sample, containing 4 µL of template DNA (40 ng), 0.75 µL of MgCl_2_ (50 mM), 1.5 µL of 10 × PCR buffer, 1.2 µL of dNTP (2.5 mM), 1.5 µL of each primer (10 mM) paired, 0.3 µL of commercial Taq DNA polymerase (1 U·µL^−1^), and nuclease-free water. The amplification program consisted of an initial denaturation step at a temperature of 94 °C for 3 min, followed by 35 cycles consisting of denaturation at 94 °C for 30 s, annealing temperature for 60 s, and extension at 72 °C for 45 s per cycle, followed by final extension, with maintenance at 72 °C for 5 min and completion of the reaction with a temperature reduction up to 10 °C until the samples were removed from the thermocycler.

After amplification, all products of the PCR-IRAP and PCR-REMAP reactions were stained with 3 µL of running buffer solution containing GelRed^®^. The amplified fragments were separated with 2% agarose gel electrophoresis in 5× TBE buffer under a constant voltage of 85 V for approximately three hours, and they were visualized with photography obtained under UV light using the Loccus (Mountain View, CA, USA) L-PIX EX 25 × 30 image capture system. The length of the amplified bands was inferred by comparison to the Invitrogen Plus 1 Kb reference ladder (Waltham, MA, USA). The electrophoretic profiles generated were used for the comparison and identification of polymorphism between the somaclones and the control plant.

### 2.4. Molecular Data Analysis and Sequencing

The agarose gels with the combinations of primers used in the study did not have a sufficient number of polymorphic bands for use in calculating a genetic diversity matrix. However, the few polymorphic bands between the somaclonal variants and the control were selected and paired-end sequenced (ACTGene https://actgene.com.br/, accessed on 1 May 2024).

The Seqassem software (version 1.0.0.0.) was used to align the sequences, and the BLASTn (Basic Local Alignment Search Tool) tool in the NCBI database (https://www.ncbi.nlm.nih.gov/, accessed on 1 May 2024) was used with complementation of the biological function.

## 3. Results

### 3.1. Evaluation of Somaclones Based on the Electrophoretic Profile

Both tools, IRAP and REMAP, generated electrophoretic profiles with multiple bands.

The combinations of primers that generated monomorphic profiles were LTR6149F + SukkulaF, LTR6150R + 3′LTRF, LTR6150R + C0795, LTR6150R + C0945, 5′LTR2R + SukkulaF, 3′LTRF + 3′LTRF, 3′LTRF + C0945, NikitaF + SukkulaF, NikitaF + C0795, and C0795 + C0945 using IRAP markers and LTR7286 + 8564 using REMAP markers.

In the other nine reactions using IRAP markers (C0795 + 3′LTR, 5′LTR2 + LTR6150, 5′LTR2 + Nikita, LTR6149 + Nikita, 5′LTR2 + 3′LTR, LTR6150 + Nikita, 3′LTR + Nikita, LTR6150 + Sukkula, and C0945 + Nikita) and reactions using REMAP markers, most gels presented monomorphic bands, so it was not possible to calculate a genetic dissimilarity matrix due to a very low number of polymorphic bands.

**Figure 1:**
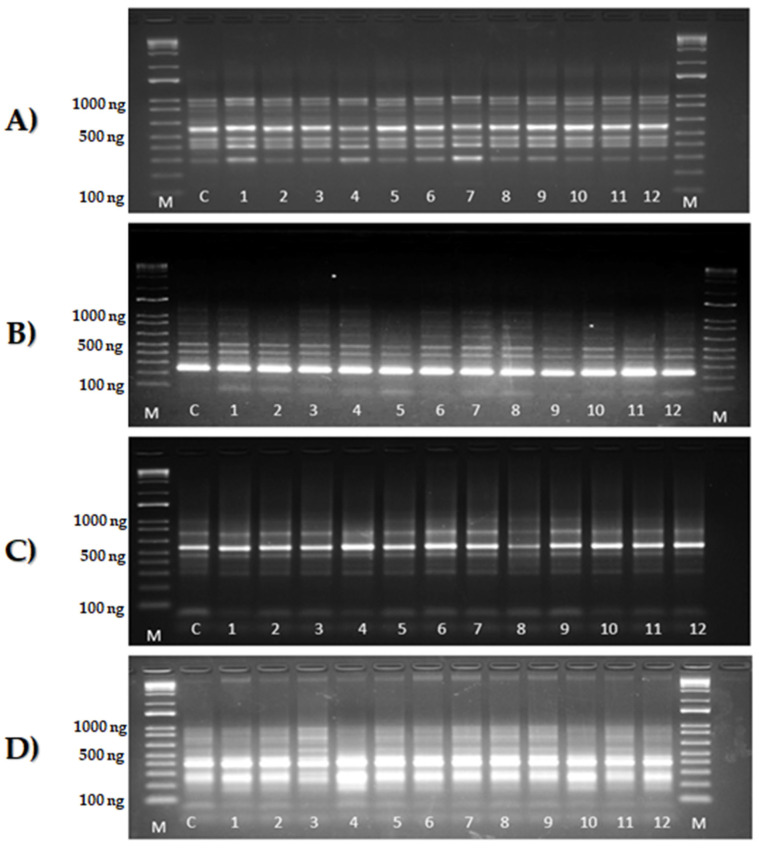
A total of 2% agarose gel with the monomorphic electrophoretic profile of the control and 12 somaclone samples derived from the cultivar Grande Naine. C = control (GN), 1–12: somaclones (Table 1). M = Ladder marker (Invitrogen^®^, Waltham, MA, USA). (**A**–**D**): IRAP and REMAP marker primer combinations (**A**) LTR7286 + 8081 (REMAP), (**B**) 5′LTR + Sukkula (IRAP), (**C**) LTR7286 + 8385 (REMAP), and (**D**) LTR7286 + 8386 (REMAP).

A profile of agarose gels using IRAP and REMAP markers is shown in Figure 1A–D.

**Figure 2:**
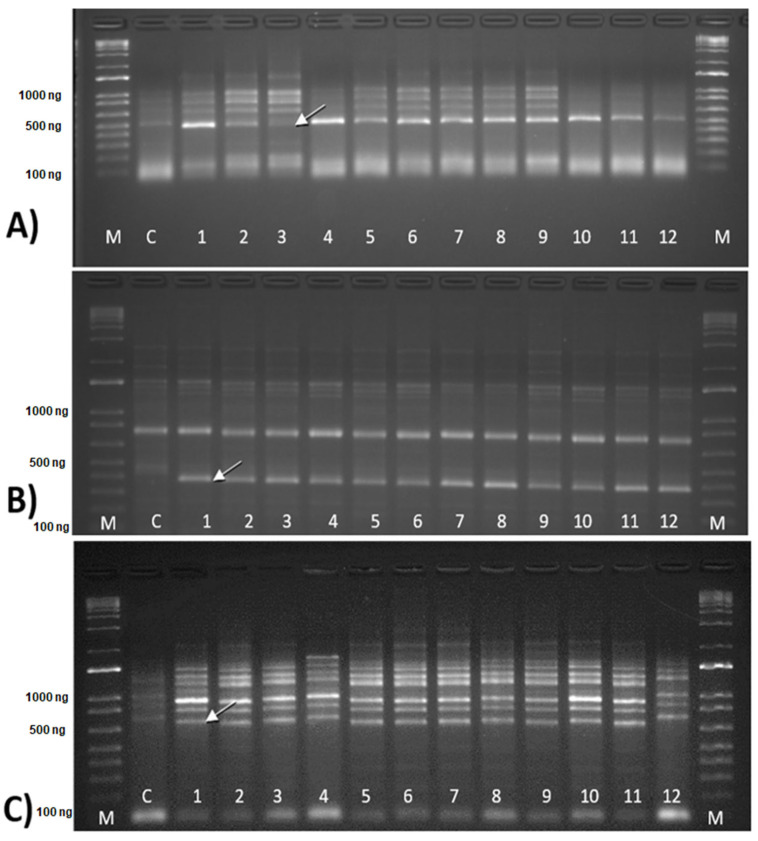
Electrophoretic profile of ‘Grande Naine’ control and somaclones (1–12) on 2% agarose gel. C = control (GN), 1–12: somaclones (Table 1). M = Ladder marker (Invitrogen^®^). (**A**) Band 1AF (white arrow), LTR6149 (T1B2P6) + Nikita (T1B2P6: 1–12), (**B**) Band 2AF (white arrow), 3′LTR (T1B2P2) + Nikita (T1B2P2: 1–11), and (**C**) Band 3AF (white arrow), Sukula + LTR6150 (T1B2P2: 1–11).

However, polymorphic bands were detected in the primer combinations, as follows: LTR6149 (T1B2P6) + Nikita, 3′LTR (T1B2P2) + Nikita, and Sukula + LTR6150 (Figure 2A–C) and chosen randomly for sequencing.

### 3.2. Analysis of Polymorphic Bands and Sequencing

From the paired combination between eight LTR primers (Table 2), 19 amplification reactions were performed using the PCR-IRAP method, of which 9 generated electrophoretic profiles that were analyzed in this study. The Nikita primer constituted five of the nine polymorphic reactions, while the 3′LTR and 5′LTR2 primers, were used in three of them.

The optimal annealing temperature of the IRAP primers ranged from 45 °C (C0795 +3′LTR) to 48.4 °C (5′LTR2 +3′LTR), averaging 46.7 °C.

The polymorphic bands presented in Figure 2 (white arrow) were sequenced (paired-end) and aligned. The result of alignment, BLASTn (https://www.ncbi.nlm.nih.gov/, accessed on 1 May 2024), and function are shown in Table 4.

## 4. Discussion

### 4.1. Evaluation of Somaclones Based on Electrophoretic Profiles

The classical genetic breeding in *Musa* spp. is based on the development of hybrid cultivars that aggregate genes that confer palatability to fruits, short stature, high production, and resistance to main biotic and abiotic factors [47,48]. However, the transfer of desired traits by sexual reproduction between diploids and triploids is a challenge posed by the absence of viable seeds in commercial triploids and parthenocarpy innate to the species. In this context, the induction of somaclonal variation in tissue culture is an important source of variability for the banana genetic breeding programs [17,21] aiming to obtain resistance to abiotic and biotic stresses in a permanent and heritable way.

In this study, we evaluated variant somaclones previously considered resistant to *Fusarium oxysporum* f.sp. *cubense* race 4 subtropical (Foc ST4), derived from the cultivar Grande Naine. Fifteen combinations of primers 9 IRAP [26,49,50] and 6 REMAP [31]; electrophoretic profiles were generated and analyzed.

Starting from the Copia type LTR of BARE-1, Sabrina, Nikita, and Sukkula families, 10 primer combinations of IRAP markers, and 1 combination for the REMAP markers generated monomorphic profiles. Among the other PCR reactions, most of the primer combinations used generated monomorphic bands, in principle, not allowing a deeper study of genetic dissimilarity between the somaclones analyzed.

Genetic somaclonal variation can sometimes involve base changes arising from variation in a single nucleotide. When evaluating somaclones derived from seven cultivars of the Cavendish subgroup (“Hsien Jen Chiao”, “Grande Naine”, “Umalag”, “Williams”, “Giant Cavendish”, “Valery”, and “Tai-Chiao N°2”– TC2), all resistant to tropical Foc race 4, Hou et al. [51] identified polymorphism using RNA sequencing, confirming the occurrence of single nucleotide variation, as well as deletions in chromosomes and chimerism.

Similar results were found by Mirani et al. [42] in screening somaclonal variants derived from two date palm cultivars (*Phoenix dactylifera*) using IRAP markers. The authors distinguished nine clonal variants according to the number of subcultures and the genotype of the Gulistan and Kashuwari cultivars in a sample of 90 individuals. Although polymorphism was detected in nine variant regenerants, 100% similarity was detected in relation to the mother plant, forming two distinct groups referring to the cultivars.

### 4.2. Evaluation of Polymorphic Bands and Sequencing

A relevant consequence of the wide distribution and adaptation of bananas to various environments is the diversity of interactions between the genotypes of edible bananas and local pathogens. The genera *Musa* and *Ensete* have narrow genetic bases for genes related to disease resistance [1], and although a plant’s immune system does not exist per se, plants have the innate ability to recognize external stressors and strategically initiate defense responses as to confer resistance or susceptibility to host plants [8].

In our study, despite the high rate of similarity between the somaclones and between them and the control plant, it was possible to identify a polymorphic segment potentially related to defense pathways. The selected bands (Figure 2A–C) blasted against the databases are all of the *Musa* genus (Table 4); however, only one of them, the 1AF band, had an interesting result regarding disease resistance, as it signals the presence of calcium-binding proteins, more precisely, calcium-binding proteins similar to kinesin-interacting Ca^2+^-binding protein (KIC). Calmodulin (CaM) is a calcium-binding protein related to motility and microtubule clustering [52], and kinesins are molecular motors involved in several cellular processes. KCBPs are calmodulin-binding proteins of the kinesin type. In this context, KIC proteins have a CaM-like sequence and compete with them for binding to KCBP [53].

The study conducted by Reddy et al. [53] describes the KIC protein, detailing its constitution and function. KIC proteins exhibit a single EF-hand binding motif and compete with calmodulin (CaM) proteins for the binding site on calmodulin-linked, kinesin-like proteins (KCBPs). Like CaM, KIC inhibits the interaction of KCBPs with microtubules as a function of Ca^2+^ concentration, interfering with trichome morphogenesis. KIC differs from CaM proteins by requiring a low concentration of Ca^2+^ for the inactivation of KCBPs. The authors also emphasize that the inhibition triggered at a low concentration of free Ca^+2^ in the cell can be interpreted as early inactivation of KCBP to signal an initial increase in the concentration of free Ca^+2^ in the formation of trichomes. Finally, the results also demonstrated that inactivation does not prevent trichome morphogenesis but results in reduced trichomes.

In the search for a correlation between microtubules and fungal development, Konzack et al. [54] characterized a kinesin superfamily protein (*kipA*) for its composition and function in *Aspergillus nidulans.* Their results showed that the kinesin motor protein isolated by them (kipA) is responsible for the growth and directional orientation of microtubules in *A. nidulans*. Therefore, Konzac et al. [54] concluded that the correct orientation of growth of the microtubes depends on the integrity of the motion protein.

With the understanding of the function of the KIC proteins, it is necessary to also understand the importance of calcium-binding proteins. Calcium-binding proteins are responsible for the interception, decoding, and amplification of the meaning of these variations in calcium concentration, enabling the necessary and sometimes specific defense responses [55,56]. Ca^+2^ ions are important secondary messengers related to numerous metabolic pathways in plants and are highly susceptible to biotic and abiotic stresses, which rapidly alter their concentration in cells [55]. The increase in Ca^+2^ concentration is the first trigger to initiate the interaction between receptors and molecular patterns activating calcium-bound proteins [57]. The ability of plants to recognize stressing agents stems from the interaction between their pattern recognition receptors (RRPs) and the molecular patterns associated with microbes or pathogens (M/PAMPs). When this relationship is established, defense strategies are initiated, and the process is referred to as immunity triggered by PAMPs [58].

Many studies that have focused on gene expression in response to biotic stresses have reported the identification of calcium-bound proteins. Meng et al. [44,59], aiming to identify genes resistant and susceptible to *Phytophthora nicotianae* in tobacco cultivars, Beihart1000-1 (BH) and Xiaohuangjin 1025 (XHJ), performed RNA sequencing, detecting 23,753 and 25,187 differentially expressed genes. In their results, in addition to resistance and disease-related proteins, four calcium-binding proteins were identified. Likewise, studies conducted by Lu et al. [56] that aimed to evaluate the role of the TaCML36 gene in the immune response of wheat to *Rhizoctonia cerealis* detected high gene expression with consequent transcription of a calcium-binding protein similar to calmodulin, whose main role is gene regulation. According to Lu et al. [56], the set of results guides the strong and positive action of the protein in the innate immunity response by modulating the expression of defense genes with possible impacts on the ethylene pathway.

Several other recent studies have demonstrated the expression of calcium-binding proteins in the resistance response of plants to the *Fusarium* spp. complex [60]. The wheat infection by *Fusarium graminearum* induces in plants resistant to ear burning the expression of HisR, a calcium-binding protein rich in histidine but with mutation represented by deletion of 752 bp at the 5′ end [61]. The results of the research by Li et al. [61] demonstrate that this protein, detected in each chromosome of the three genomes (A, B, C) of wheat, encodes 260 amino acids, and the intensity of its expression is positively correlated with plant resistance. The first study of differentially expressed genes related to tomato fusariosis under field conditions was carried out by Ribeiro et al. [57]. In this study, the authors observed a positive correlation between the intensity of infection and the expression of transcription factor WRKY41 and calcium-binding genes, CBEF, both involved in innate resistance to *Fusarium oxysporum* and *F. incarnatum-equiseti*.

Calcium-binding proteins also have a close relationship with defense responses to abiotic stress, such as water stress [62], chemical stress from pesticide application [63], and salt stress [64]. The relationship between these proteins and abiotic stresses is well listed in Zeng et al. [65].

In our work, the markers IRAP and REMAP were important in the detection of polymorphism, enabling the identification of a polymorphic fragment involved with plant defense response. It is worth mentioning that Embrapa Mandioca e Fruticultura is one of the most renowned institutions carrying out the genetic breeding program of bananas worldwide and one of the most representative in Latin America, with more than 400 accessions in its collection and new improved diploids and new hybrids constantly being developed through its ongoing breeding program. Being able to explore new markers and bioinformatic tools in order to contribute to its ongoing genetic banana breeding program can remarkably accelerate the obtainment of more resistant and productive banana varieties.

Our work demonstrates that IRAP and REMAP markers, along with sequencing and bioinformatics, are interesting tools for identifying polymorphism of interest when it comes to somaclonal variants. Many more bands of the somaclones derived from the same mother plant should be sequenced, but the initial results demonstrate that the band identified in our work may be used in marker-assisted selection in banana breeding programs in the seedling phase, given new primer designs and extensive field validations.

## 5. Conclusions

Biotechnological advances are an important driving factor in the genetic improvement of plants, aiming at the rapid adaptation of cultivars and commercial varieties to biotic and abiotic stresses. The use of IRAP and REMAP markers in a banana breeding programs where activities are ongoing in the search for the development of varieties more resistant/tolerant to the main biotic and abiotic factors is of paramount importance when it comes to MAS to accelerate results. In the case of *Musa* spp., given the inherent parthenocarpy of the species along with the narrow genetic base, the use of these tools is of outmost necessity. With the use of molecular markers based on retrotransposons, we confirmed the desirable genomic stability in the somaclones that were induced by mutation. However, the markers used made it possible to detect a polymorphic band that, when sequenced, identified variants in the somaclones, a gene related to a calcium-binding protein. With the continuity of the research, it will be possible to evaluate the resistance, in the field, of the variant somaclones. It will also be possible to measure the effectiveness and scope of the marker identified in this study in assisted selections for resistance to Foc ST4 in the *genus* Musa. Therefore, the results presented show that biotechnology combined with bioinformatic tools enable better design of crop breeding strategies within a scenario that aims to aggregate information to contribute to a more sustainable and promising production chain and agribusiness of this important fruit.

## Figures and Tables

**Table 1:** List of evaluated somaclones previously considered resistant to Foc ST4 derived from the cultivar Grande Naine (*Musa* spp.; Cavendish—AAA) and degree of resistance.

Treatment		Origin Research Center	Degree of Resistance
C	Control (‘Grande Naine’)	Embrapa Mandioca e Fruticultura	MR
1	Embrapa 14—T1B2P2	Embrapa Mandioca e Fruticultura	HR
2	Embrapa 14—T1B2P3	Embrapa Mandioca e Fruticultura	HR
3	Embrapa 14—T1B2P6	Embrapa Mandioca e Fruticultura	HR
4	Embrapa 15—T2B1P1	Embrapa Mandioca e Fruticultura	HR
5	Embrapa 15—T2B1P5	Embrapa Mandioca e Fruticultura	HR
6	Embrapa 15—T2B1P7	Embrapa Mandioca e Fruticultura	HR
7	Embrapa 16—T3B2P4	Embrapa Mandioca e Fruticultura	HR
8	Embrapa 16—T3B2P6	Embrapa Mandioca e Fruticultura	HR
9	Embrapa 16—T3B2P8	Embrapa Mandioca e Fruticultura	HR
10	Embrapa 17—T4B1P2	Embrapa Mandioca e Fruticultura	R
11	Embrapa 17—T4B1P4	Embrapa Mandioca e Fruticultura	R
12	Embrapa 17—T4B1P6	Embrapa Mandioca e Fruticultura	R

MR: Moderately resistant; HR: highly resistant; R: resistant.

**Table 2:** Detail of IRAP primers used in somaclones resistant to Foc ST4 derived from the cultivar Grande Naine, indicating orientation, origin, sequence, and referenced authors.

Primers	Family	Sequence (5′-3′)	Reference
5′LTR2 ←	Bare 1	5′-ATCATTGCCTCTAGGGCATAATTC-3′	[26]
3′LTR →	Bare 1	5′-TGTTTCCCATGCGACGTTCCCCAACA-3′	[26]
Sukkula	Sukkula	5′-GATAGGGTCGCATCTTGGGCGTGAC-3′	[26]
Nikita →	Nikita	5′-CGCATTTGTTCAAGCCTAAACC-3′	[26]
LTR6149 →	Bare 1	5′-CTCGCTCGCCCACTACATCAACCGCGTTTATT-3′	[26]
LTR6150 ←	Bare 1	5′-CTGGTTCGGCCCATGTCTATGTATCCACACATGTA-3′	[26]
C0795	Bare 1	5′-TCCCATGCGACGTTCCCC-3′	[46]
C0945	Sabrina	5′-GCAAGCTTCCG TTCCGC-3′	[46]

→: Forward; ←: Reverse.

**Table 3:** Detailed list of microsatellite primers (SSRs) and retrotransposon LTR [31], used in somaclones resistant to Foc ST4 derived from the cultivar Grande Naine, with orientation and nucleotide sequence.

Primers	Sequence (5′-3′)	Reference
SSR		[31]
8081 →	(GA)9C	[31]
8082 →	(CT)9G	[31]
8385 →	(CAC)7G	[31]
8386 →	(GTG)7C	[31]
8387 →	(CA)10G	[31]
8564 →	(CAC)T7	[31]
8565 →	GT(CAC)7	[31]
LTR		
LTR7286 ←	GGAATTCATAGGATGGATAATAAACGATTATC	[31]

Arrows to the right (→)—Forward and arrows to the left (←)—Reverse.

**Table 4:** Information of the polymorphic bands sequenced based on polymorphism between control and possible somaclones, primers used, alignment of the sequences in the Seqassem software, approximate size of the bands, and function according to the BLAST/NCBI algorithm.

Name	Seqassem	Size (bp)	BLAST: ID	Function
1AF Primer forward: LTR6149 (T1B2P6) 1AR Primer reverse: Nikita	>CTGCAAACACGACCTCCCTCTCGATCGCCTTCTCCAGCCACGCCTCGGCGTCCGCCATCATCTCCGGGCTCAGCCTCACCATCAGCACGCAGAACTCCTTCTCGTCGAGCGCCCCGTCCCCGTCCATGTCCCCTTCCCTCACCATCGCCGCCGCATCCTCCGCCGTCATCCCCGCCATCCCCAGCGCCGCCGCGTTCCTCCTCAGGCTCTCCGGCGTTATGACCCCCCTCCCGGGCTCCGCCAGCAACCGGAAGCCCCCGCaCAGCTCCGACACGAACTGCTCCGCTTCCAGCCTCTCGGCCATCACCGGCACCAAGTCCTCGTACTCCTCCGACTCCGTCGCAGCTGCGTGCTTCTCTTCCTCCATCGCCAGACCTTCCAAGTCTCTGTTGATGCTGCARARATGGTTTAGGCTTG	Approximately 400	*Musa acuminata* subsp. *malaccensis* isolate AA chromosome 15 Sequence ID: XM_065104944	calcium-binding protein KIC-like
2AF Primer forward: 3′LTR (T1B2P2) 2AR Primer reverse: Nikita	>GGTGTTAACTATTACTATATAGTAAATAGGGCTCTCGAACAACACTTGAGGAATCACTCACTCCTACTTAGCTACTATCTGAACTCACTCCTACAGTTGCTCACAAGAACCGGAGCAGTCAAACTAGGGACAAGAACAAGAAAAGGACTACTATCTTCTTGCCAACCCTTAATACAGGACTTGTAATAACTCTGGAACCTCTCTCTCCACTACTTGGGAAAGCCAATATTAGATGAGATGAGCCTCATTCCGCAAGTAAGAATGAAGTTAGGGTACTAGAATTAGCAGTGCCCCCGGGCATCTAGAATTAGCAGTGCTAGTTATAAAGTAAGG	Approximately 300	*Musa acuminata* subsp. *malaccensis* strain Doubled-haploi. Score: 544 Evalue: 8.3 × 10^−150^ Accession:HG996478.1	
3AF Primer forward: Sukkula (T1B2P2) 3AR Primer reverse: LTR6150	>ACCAGTAGCAGCCCATTCAGACCCAGATTTCTTGGCCGGCGGTGGCATTGCCCAGGAGCTCAGGAAAGCTAGGCAGTGCAATAGCCCGGGTCAGGGGGTGGTACCACCTGGGCTTAGTCTCCGAGCAAGACTGGGCAGTGGTACCACTTGGGCTAAATCTCTGAGCGAGACTGGGCAGTGGTACCGCCCCTATCAGGCAGTGGTACTGCCTGAGCTCGGTCTCCGAGAGGTAGTACTGCCCAGTTATATTGGTAGTACCGCCAGGACCTCGGAAATCTAGGAGATGACACATTTGAGCTCCAAATTCAAATCAGTTGGGGGCTATATGTAATATCCCTCACTTTTAAAAATTTATTAATAAGGATTTATATGTAAATTGGAGGACCTATATGTAAATATAGAAATTTTAAGGATTAAACTGTTAAGTTGCAAAAAGAAAAAAAAATTAAAGAAAACCGAAGAGGGAAAAGAAGAAAGAAAGAAGAGGGAGAAGAGAAGAAAAGAAGAAGAAGAAGAGAGGGAAGAGGGAGCGGGATGAGAAAGATAGCTGCAGTAGATAGGGCTGCAGCGCCTCTGTTTCGTGTAGGAGGAAACARAGGAGTACATGTGTGGATACATASACATGGGCSGAACCASACAAA	Approximately 600	*Musa acuminata* subsp. *malaccensis* isolate AA chromosome 14 GenBank: CP126383.1	

## Data Availability

Data will be made available upon request.

## References

[1] Rijzaani H., Bayer P.E., Rouard M., Doležel J., Batley J., Edwards D. (2022). The pangenome of banana highlights differences between genera and genomes. Plant Genome.

[2] Fu N., Ji M., Rouard M., Yan H.-F., Ge X.-J. (2022). Comparative plastome analysis of Musaceae and new insights into phylogenetic relationships. BMC Genom..

[3] FAO—Food and Agriculture Organization of the United Nations (2024). Faostat. https://www.fao.org/faostat/en/#data/QCL/visualize.

[4] Bebber D.P. (2023). The long road to a sustainable banana trade. Plants People Planet.

[5] Castelan F.P., Saraiva L.A., Lange F., De Lapeyre de Bellaire L., Cordenunsi B.R., Chillet M. (2012). Effects of Black Leaf Streak disease and Sigatoka disease on fruit quality and maturation process of bananas produced in the subtropical conditions of southern Brazil. Crop Prot..

[6] Ocimati W., Bouwmeester H., Groot J.C.J., Tittonell P., Brown D., Blomme G. (2019). The risk posed by Xanthomonas wilt disease of banana: Mapping of disease hotspots, fronts and vulnerable landscapes. PLoS ONE.

[7] Simbare A., Sane C.A.B., Nduwimana I., Niyongere C., Omondi B.A. (2020). Diminishing farm diversity of East African highland bananas in Banana Bunchy Top Disease outbreak areas of Burundi—The effect of both disease and control approaches. Sustainability.

[8] Segura-Mena R.A.S., Stoorvogel J.J., García-Bastidas F., Salacinas-Niez M., Kema G.H.J., Sandoval J.A. (2021). Evaluating the potential of soil management to reduce the effect of *Fusarium oxysporum* f. sp*. cubense* in banana (Musa AAA). Eur. J. Plant Pathol..

[9] Ploetz R.C. (2015). Management of Fusarium wilt of banana: A review with special reference to tropical race 4. Crop Prot..

[10] Warman N.M., Aitken E.A.B. (2018). The movement of *Fusarium oxysporum* f. sp. *cubense* (Sub-Tropical Race 4) in susceptible cultivars of banana. Front. Plant Sci..

[11] Pegg K.G., Coates L.M., O’Neill W.T., Turner D.W. (2019). The epidemiology of Fusarium Wilt of banana. Front. Plant Sci..

[12] Ghag S.B., Shekhawat U.K.S., Ganapathi T.R. (2015). Fusarium wilt of banana: Biology, epidemiology and management. Int. J. Pest Manag..

[13] Dita M.A., Barquero M., Heck D., Mizubuti E.S.G., Staver C.P. (2018). Fusarium Wilt of banana: Current knowledge on epidemiology and research needs toward sustainable disease management. Front. Plant Sci..

[14] Staver C., Pemsl D.E., Scheerer L., Perez-Vicent L.F., Dita M. (2020). Ex ante assessment of returns on research investments to address the impact of Fusarium wilt tropical race 4 on global banana production. Front. Plant Sci..

[15] Ploetz R.C. (2006). Fusarium Wilt of banana is caused by several pathogens referred to as *Fusarium oxysporum* f. sp. *cubense*. Phytopathology.

[16] Peredo E.L., Arroyo-García R., Revilla M.Á. (2009). Epigenetic changes detected in micropropagated hop plants. J. Plant Physiol..

[17] Ferreira M.D.S., Rocha A.D.J., Nascimento F.D.S., Oliveira W.D.D.S., Soares J.M.D.S., Rebouças T.A., Morais Lino L.S., Haddad F., Ferreira C.F., Santos-Serejo J.A.D. (2023). The role of somaclonal variation in plant genetic improvement: A systematic review. Agronomy.

[18] Bairu M.W., Aremu A.O., Van Staden J. (2011). Somaclonal variation in plants: Causes and detection methods. Plant Growth Regul..

[19] Ferreira M.d.S., de Moura É.R., Lino L.S.M., Amorim E.P., dos Santos-Serejo J.A., Haddad F. (2020). Selection of somaclonal variants of the cultivar ‘Prata-Anã’ for resistance to *Fusarium oxysporum* f. sp*. cubense* race 1. Rev. Bras. Frutic..

[20] Maciejewski P., Ramm A., Moreira R.M., Oliveira B.A.S., Mattos M.G., Assis A.M., Schuch M.W. (2020). In vitro subcultures of blueberry cultivars. Braz. J. Dev..

[21] Rebouças T.A., Rocha A.J., Cerqueira T.S., Adorno P.R., Barreto R.Q., Ferreira M.S., Lino L.S.M., Amorim V.B.O., Santos-Serejo J.A., Haddad F. (2021). Pre-selection of banana somaclones resistant to *Fusarium oxysporum* f. sp. *cubense*, subtropical race 4. Crop Prot..

[22] Oh T.J., Cullis M.A., Kunert K., Engelborghs I., Swennen R., Cullis C.A. (2007). Genomic changes associated with somaclonal variation in banana (*Musa* spp.). Physiol. Plant..

[23] Roux N., Chase R., Van Den Houwe I., Chao C.-P., Perrier X., Jacquemoud-Collet J.P., Sardos J., Rouard M., Sivasankar S., Ellis N., Jankuloski L., Ingelbrecht I. (2021). Somaclonal variation in clonal crops: Containing the bad, exploring the good. Mutation Breeding, Genetic Diversity and Crop Adaptation to Climate Change.

[24] Arvas Y.E., Kocaçalişkan İ., Ordu E., Erişen S. (2022). Comparative retrotransposon analysis of mutant and non-mutant rice varieties grown at different salt concentrations. Biotechnol. Biotechnol. Equip..

[25] Häkkinen M., Teo C.H., Othman Y.R. (2007). Genome constitution for *Musa beccarii* (Musaceae) varieties. J. Syst. Evol..

[26] Teo C.H., Tan S.H., Ho C.L., Faridah Q.Z., Othman Y.R., Heslop-Harrison J.S., Kalendar R.N., Schulman A.H. (2005). Genome constitution and classification using retrotransposon-based markers in the orphan crop banana. J. Plant Biol..

[27] De Carvalho Santos T.T., de Oliveira Amorim V.B., dos Santos-Serejo J.A., Silva Ledo C.A., Haddad F., Ferreira C.F., Amorim E.P. (2019). Genetic variability among autotetraploid populations of banana plants derived from wild diploids through chromosome doubling using SSR and molecular markers based on retrotransposons. Mol. Breed..

[28] Saraswathi M.S., Uma S., Ramaraj S., Durai P., Mustaffa M.M., Kalaiponmani K., Chandrasekar A. (2020). Inter retrotransposon based genetic diversity and phylogenetic analysis among the Musa germplasm accessions. J. Plant Biochem. Biotechnol..

[29] Du C., Swigoňová Z., Messing J. (2006). Retrotranspositions in orthologous regions of closely related grass species. BMC Evol. Biol..

[30] Kalendar R., Flavell A.J., Ellis T.H.N., Sjakste T., Moisy C., Schulman A.H. (2011). Analysis of plant diversity with retrotransposon-based molecular markers. Heredity.

[31] Kalendar R., Grob T., Regina M., Suoniemi A., Schulman A. (1999). IRAP and REMAP: Two new retrotransposon-based DNA fingerprinting techniques. Theor. Appl. Genet..

[32] Jing H., Esfandani-Bozchaloyi S. (2022). Genetic diversity and gene-pool of *Medicago polymorpha* L. based on retrotransposon-based markers. Caryologia.

[33] Koroluk A., Paczos-Grzęda E., Sowa S., Boczkowska M., Toporowska J. (2022). Diversity of Polish Oat Cultivars with a Glance at Breeding History and Perspectives. Agronomy.

[34] Dongare M.D., Alex S., Soni K.B., Sindura K.P., Nair D.S., Stephen R., Jose E. (2023). Cross-species transferability of IRAP retrotransposon markers and polymorphism in black pepper (*Piper nigrum* L.). Genet. Resour. Crop Evol..

[35] Razi M., Amiri M.E., Darvishzadeh R., Doulati Baneh H., Alipour H., Martínez-Gómez P. (2020). Assessment of genetic diversity of cultivated and wild Iranian grape germplasm using retrotransposon-microsatellite amplified polymorphism (REMAP) markers and pomological traits. Mol. Biol. Rep..

[36] Villano C., Corrado G., Basile B., Di Serio E., Mataffo A., Ferrara E., Aversano R. (2023). Morphological and Genetic Clonal Diversity within the ‘Greco Bianco’ Grapevine (*Vitis vinifera* L.) Variety. Plants.

[37] Minaei S., Mohammadi S.A., Sabouri A., Dadras A.R. (2022). High genetic diversity in *Aegilops tauschii* Coss. accessions from North Iran as revealed by IRAP and REMAP markers. J. Genet. Eng. Biotechnol..

[38] Holasou H.A., Rahmati F., Rahmani F., Imani M., Talebzadeh Z. (2019). Elucidate Genetic Diversity and Population Structure of Bread Wheat (*Triticum aestivum* L.) Cultivars Using IRAP and REMAP Markers. J. Crop Sci. Biotechnol..

[39] Valadez-Moctezuma E., Arroyo-Álvarez E., Samah S. (2018). Activation of transposable elements and insertional polymorphism in Opuntia offspring as assessed by inter-retrotransposon amplified polymorphism markers. Plant Biosyst.-Int. J. Deal. All Asp. Plant Biol..

[40] Rashid K., Othman R.Y., Ali B.S.B.K.S., Yusof Y.M., Nezhadahmadi A. (2014). The Aplication of IRAP Markers in the Breeding of Papaya (*Carica papaya* L.). Indian J. Sci. Technol..

[41] Shingote P.R., Amitha Mithra S.V., Sharma P., Devanna N.B., Arora K., Holkar S.K., Khan S., Singh J., Kumar S., Sharma T.R. (2019). LTR retrotransposons and highly informative ISSRs in combination are potential markers for genetic fidelity testing of tissue culture-raised plants in sugarcane. Mol. Breed..

[42] Mirani A.A., Teo C.H., Markhand G.S., Abul-Soad A.A., Harikrishna J.A. (2000). Detection of somaclonal variations in tissue cultured date palm (*Phoenix dactylifera* L.) using transposable element-based markers. Plant Cell Tissue Organ Cult..

[43] Nasri F., Zakizadeh H., Vafaee Y., Mozafari A.A. (2022). In vitro mutagenesis of *Chrysanthemum morifolium* cultivars using ethylmethanesulphonate (EMS) and mutation assessment by ISSR and IRAP markers. Plant Cell Tissue Organ Cult..

[44] Muhammad A.J., Othman F.Y. (2005). Characterization of fusarium wilt-resistant and fusarium wilt-susceptible somaclones of banana cultivar rastali (*Musa* AAB) by random amplified polymorphic DNA and retrotransposon markers. Plant Mol. Biol. Rep..

[45] Ferreira C.F., Gutierrez D.L., Kreuze J.F., Iskra-Caruana M.L., Chabannes M., Barbosa A.C.O., Santos T.A., Silva A.G.S., Santos R.M.F., Amorim E.P. (2019). Rapid plant DNA and RNA extraction protocol using a bench drill. Genet. Mol. Res..

[46] Baumel A., Ainouche M., Kalendar R., Schulman A.H. (2002). Retrotransposons and genomic stability in populations of the young allopolyploide species *Spartina anglica* C.E. Hubbard (Poaceae). Mol. Biol. Evol..

[47] Shepherd K. (1987). Banana Breeding—Past and Present. Acta Hortic..

[48] Amorim E.P., Amorim V.B.O., Silva S.O., Pillay M., Michael Pillay A.T. (2011). Quality improvement of cultivated Musa. Banana Breeding: Progress and Challenges.

[49] Saraswathi M.S., Uma S., Prasanya Selvam K., Ramaraj S., Durai P., Mustaffa M.M. (2011). Assessing the robustness of IRAP and RAPD marker systems to study intra-group diversity among Cavendish (AAA) clones of banana. J. Hortic. Sci. Biotechnol..

[50] Shelke R.G., Das A.B. (2015). Analysis of genetic diversity in 21 genotypes of indian banana using RAPDs and IRAPs markers. Proc. Natl. Acad. Sci. India Sect. B Biol. Sci..

[51] Hou B.H., Tsai Y.H., Chiang M.H., Tsao S.M., Huang S.H., Chao C.P., Chen H.M. (2022). Cultivar-specific markers, mutations, and chimerisim of Cavendish banana somaclonal variants resistant to *Fusarium oxysporum* f. sp. *cubense* tropical race 4. BMC Genom..

[52] Kao Y.L., Deavours B.E., Phelps K.K., Walker R.A., Reddy A.S.N. (2000). Bundling of microtubules by motor and tail domains of a kinesin-like calmodulin-binding protein from Arabidopsis: Regulation by Ca^2+^/calmodulin. Biochem. Biophys. Res. Commun..

[53] Reddy V.S., Day I.S., Thomas T., Reddy A.S.N. (2004). KIC, a novel Ca^2+^ binding protein with one EF-hand Motif, interacts with a microtubule motor protein and regulates trichome morphogenesis. Plant Cell.

[54] Konzack S., Rischitor P.E., Enke C., Fischer R. (2005). The role of the kinesin motor KipA in microtubule organization and polarized growth of *Aspergillus nidulans*. Mol. Biol. Cell.

[55] Lecourieux D., Ranjeva R., Pugin A. (2006). Calcium in plant defence-signalling pathways. New Phytol..

[56] Lu L., Rong W., Zhou R., Huo N., Zhang Z. (2019). TaCML36, a wheat calmodulin-like protein, positively participates in an immune response to *Rhizoctonia cerealis*. Crop J..

[57] Ribeiro J.A., Albuquerque A., Materatski P., Patanita M., Varanda C.M.R., Félix M.d.R., Campos M.D. (2022). Tomato response to *Fusarium* spp. Infection under field conditions: Study of potential genes involved. Horticulturae.

[58] Postel S., Kemmerling B. (2009). Plant systems for recognition of pathogen-associated molecular patterns. Semin. Cell Dev. Biol..

[59] Meng H., Sun M., Jiang Z., Liu Y., Sun Y., Liu D., Jiang C., Ren M., Yuan G., Yu W. (2021). Comparative transcriptome analysis reveals resistant and susceptible genes in tobacco cultivars in response to infection by *Phytophthora nicotianae*. Sci Rep..

[60] Liu M., Sui Y., Yu C., Wang X., Zhang W., Wang B., Yan J., Duan L. (2023). Coronatine-Induced Maize Defense against Gibberella Stalk Rot by Activating Antioxidants and Phytohormone Signaling. J. Fungi.

[61] Li S., Ramakrishnan M., Vinod K.K., Kalendar R., Yrjälä K., Zhou M. (2020). Development and deployment of high-throughput retrotransposon-based markers reveal genetic diversity and population structure of asian bamboo. Forests.

[62] Gong X., Xu Y., Li H., Chen X., Song Z. (2022). Antioxidant activation, cell wall reinforcement, and reactive oxygen species regulation promote resistance to waterlogging stress in hot pepper (*Capsicum annuum* L.). BMC Plant Biol..

[63] An M., Zhou T., Guo Y., Zhao X., Wu Y. (2019). Molecular regulation of host defense responses mediated by biological anti-TMV agent ningnanmycin. Viruses.

[64] Bao Y., Chen C., Fu L., Chen Y. (2021). Comparative transcriptome analysis of *Rosa chinensis* ‘Old Blush’ provides insights into the crucial factors and signaling pathways in salt stress response. Agronomy.

[65] Zeng H., Xu L., Singh A., Wang H., Du L., Poovaiah B.W. (2015). Involvement of calmodulin and calmodulin-like proteins in plant responses to abiotic stresses. Front. Plant Sci..

